# ‘It gives you the skills of how you can cope’: Exploring the self‐reported experience of patients receiving in‐centre haemodialysis on participating in chosen art activities

**DOI:** 10.1111/hex.13337

**Published:** 2021-08-11

**Authors:** Francesca Taylor, Vari M. Drennan, Marie‐Louise Turner, Jeunita Jones, Joyce Popoola

**Affiliations:** ^1^ Centre for Health and Social Care Research Joint Faculty of Kingston University and St. George's University of London London UK; ^2^ Department of Nephrology and Transplantation St. George's University Hospitals NHS Foundation Trust London UK; ^3^ Department of Biomedical Sciences and Education St. George's University of London London UK

**Keywords:** art, kidney failure, patient preference, qualitative research, renal dialysis, self‐concept

## Abstract

**Background:**

Increasing numbers of patients are receiving dialysis, particularly in high‐income countries. Patients receiving haemodialysis often experience fatigue, anxiety, depression and boredom. It is suggested that arts activities could have a therapeutic effect.

**Objective:**

This study aimed to explore patients' perspectives of participating while on dialysis in chosen arts and creative living activities provided by tutors at the bedside.

**Design:**

Qualitative semi‐structured interviews in the interpretive tradition were conducted, with thematic analysis.

**Setting and Participants:**

Fifteen patients of different ages, genders and ethnicities who participated in an arts activity while receiving haemodialysis in an inner‐city dialysis unit in England were included in this study.

**Results:**

Participants reported positive experiences of engaging in art activities. Their views on the value of the activities were grouped into five themes: diversion from receiving haemodialysis, a sense of achievement, contribution to a more positive self‐identity, increased confidence and motivation and a therapeutic talking relationship. Participants suggested that patient peer promotion of the activities could increase uptake, with patient choice of activity seen as important.

**Conclusions:**

Participation in a chosen arts activity while receiving haemodialysis was perceived by patients to have positive psychosocial effects. We theorize three potential explanatory mechanisms for these effects: That the experience of participating in the activities engendered positive psychological states of ‘being in the flow’; enhanced self‐esteem to add to personal coping mechanisms; and offered additional facets to the patient's identity that countered the stigmatizing effect of receiving dialysis.

**Patient or Public Contribution:**

Patients and public representatives advised on the design, research methods and tools.

## 1 INTRODUCTION

Patients with end‐stage kidney disease (ESKD) experience an irreversible loss of kidney function, requiring kidney replacement therapy (KRT) by dialysis or transplantation. Kidney transplantation provides the best quality of life, while dialysis is used as an interim measure or indefinitely for those unsuitable for transplantation. About 0.1% of the global population have ESKD, with most people on KRT living in high‐income countries.^1^ Increasing numbers of patients are in need of KRT,^1^ with additional demand for dialysis associated with coronavirus disease 2019 (COVID‐19) patients.^2^ In 2018, about 26,000 adult patients received dialysis in the United Kingdom, with most patients (83%) receiving haemodialysis (HD) in a hospital or satellite dialysis unit.^3^ People of Black and minority ethnicities, those aged older than 65 years and males are overrepresented in this patient group.^3^ Patients attend a dialysis unit usually three times a week for 4–5 h at a time.^3^ Patients are connected to a dialysis machine throughout. A major challenge for patients is the considerable impact on their daily lives. Patients often experience fatigue, low mood, anxiety, depression and boredom, in addition to medical complications due to the limitations of the dialysis in serving renal functions.^4^ Many people on dialysis are known to experience a poor health‐related quality of life.^5^

Over the last 20 years, interest has grown internationally in determining the benefits that participation in the arts, that is, visual arts, dance, film, literature, music, singing, crafts and online arts, may bring to well‐being and quality of life.^6^ Some Western European countries, such as Ireland and Norway, have policies that increase the contribution of art and culture to health and well‐being.^6^ In the United Kingdom, a parliamentary group has advocated for research into the potential health benefits of arts for people living with chronic diseases.^7^ There is limited evidence assessing the benefits or otherwise of using creative arts in hospitals to improve the quality of life for patients receiving HD. A systematized literature search^8^ was undertaken of six databases (PubMed, Web of Science—Medicine, Web of Science—Arts and Humanities, Scopus, Art and Architecture Source, Google Scholar) for English‐language papers from the date of commencement of the database until 1 September 2019, later updated to 1 December 2020. Key words were used in the search (dialysis, HD, renal dialysis, arts, crafts, arts activities), combined with follow‐up of authors and references in identified papers, and forward citation searching. Seven peer‐reviewed publications were found reporting on evaluations of participatory arts activities for adult patients receiving in‐centre HD.^9^, ^10^, ^11^, ^12^, ^13^, ^14^, ^15^ Three of the papers were from the United States, two from England and one each from Ireland and Spain. The papers reported on different types of arts activities: single art activity (drawing),^9^, ^10^ two activities (creative writing and visual art),^15^ multiple activities (e.g., printmaking, mosaic making, crocheting)^12^, ^13^ and mixed active and passive activities (e.g. mandalas qmaking alongside clown visits).^10^, ^14^ Five studies involved group activities,^9^, ^11^, ^12^, ^13^, ^14^ and two studies involved individual activities.^10^, ^15^ The research methods were varied: an individual patient case study,^11^ patient interviews by purposive sampling,^9^, ^12^, ^13^ observational studies using survey instruments before and after a mixed arts programme^11^, ^14^ and mixed methods including a pilot randomized control trial and qualitative interviews.^15^ The mixed arts programme study, which was performed in the United States with 46 patients, reported significantly improved scores on the quality‐of‐life domains described as social functioning and bodily pain, for patients who had high rates of participation.^11^ The Spanish study, which included 41 patients participating in a mixed arts programme, found no statistical difference in anxiety or quality‐of‐life measures after the programme.^14^ All seven published studies reported, mostly briefly, or on the basis of professional views or reports of health professionals, that the arts programme was positively viewed by participants. Our analysis of the five studies^9^, ^10^, ^12^, ^13^, ^14^ that reported patients' reasons for the positive comments found that helping pass time and alleviating the boredom of receiving HD were the most frequently mentioned reasons. Although it is hypothesized that these activities improve mood and well‐being, there is limited evidence of effect, particularly from the patient perspective,^15^ or explanatory theories as to the mechanism of the effect.

This paper adds to the existing literature by presenting data from the perspective of patients on HD of their broad experience of participating in an arts programme in the United Kingdom, which offered choice from a range of activities and individual bedside tutorship during dialysis. This contrasts with previous studies focused on asking patients or professionals if their arts programme participation was a positive experience or not and on programmes with no choice (or only two options in the study reported by Carswell et al.^15^) and group tutorship. It is not known how many arts programmes are run in the 71 adult UK dialysis units, but a survey of 17 units found that few patients had the opportunity to participate in arts and crafts activities, although 26% of patients reported that they were interested in participating.^16^ In one inner‐city English NHS Hospital Trust, a hospital charity has provided an arts and creative living activities programme delivered by tutors at the bedside since 2016 for patients on HD. Patients were offered, unusually for these types of programmes, a wide choice of arts and creative living activities, including drawing, painting, sculpture, creative writing, languages and IT/screen‐based skills. Between July 2018 and January 2020, 127 of the 294 patients receiving HD^4^ engaged in an activity at least once. We investigated the research question from the patient perspective: what has been the patient experience, both positive and negative, of participating in a chosen arts and creative living activity/activities while receiving HD?

## 2 METHODS

### 2.1 Study design and setting

A qualitative design was used in the interpretive tradition,^17^ enabling exploration of patients' perceptions and opinions and consideration of context in understanding their experiences. Guidance for ensuring quality when undertaking qualitative research^18^ was used to assess validity and reliability in designing an appropriate methodological approach, including the interview protocol, the trustworthiness of the data collection and analysis and the extent of reflexivity. Eight patient representatives from an established patient and public involvement and engagement research expert group and from a kidney charity, volunteered to work with researchers, on a partnership basis,^19^ to develop the study. They were closely involved in developing the study design, the data collection methods and tools and the data analysis approach. The study setting was an NHS Hospital Trust with an in‐hospital unit and three outlying centres providing HD.

### 2.2 Participants

Eligibility criteria specified consenting adult patients, aged 18 years or older, who had engaged in an arts or creative living activity while receiving HD in the hospital trust in the previous 15 months. Patients were excluded if they were not clinically stable and well enough to take part or lacked capacity to provide informed consent. Sampling was purposive, designed to provide diversity in terms of age, gender, ethnicity, dialysis unit, activities experienced and extent of participation. The arts programme coordinator identified every second patient from their list of patients receiving HD in the programme and gave these details to the clinical team. The clinical team identified patients who fulfilled the eligibility criteria. A member of the clinical team made the initial enquiry about participation in the study and whether eligible patients were willing to have their contact details passed to a study researcher. Only patients indicating potential interest in participation were sent or provided with a consent form and study information sheet. This provided an explanation about the study and that they would be contacted a week later by a study researcher, which would give them an opportunity to ask any questions. They could choose to be interviewed by telephone or face to face while present in their dialysis unit and at a date/time of their choice. Four of the patients introduced to the researcher withdrew before consenting due to ill health. Recruitment continued until it was judged that data saturation in data collection had been reached, that is, when no new views or perceptions seemed to be elicited in interviews and data replication occurred.^20^

### 2.3 Data collection

Semi‐structured interviews were conducted with open‐ended questions and supplementary prompts to allow the key areas of interest to be explored without being prescriptive about content.^21^ A topic guide was developed based on the study questions and informed by discussions with PPIE representatives. A key topic that they suggested for inclusion was how and when the offer of participating in an arts or creative living activity was made to patients, hypothesising that this may influence the patient experience. Other questions included participants' motivations for taking up an activity; attitudes and feelings about participation; any perceived changes in mood and well‐being associated with undertaking the activity; and any suggested improvements. Discussion with the PPIE representatives also helped the research interviewer to reflect on how their background and experience could influence the data collection. Thirteen participants were interviewed face to face in their dialysis unit while receiving HD. One participant was interviewed by telephone and one participant was interviewed at the renal outpatient department of the study site. Interviews lasted between 18 and 52 min (mean: 29), were audio‐recorded with permission and transcribed. Field notes were taken to record interviewer observations, analytical thoughts and issues raised. Interviews were undertaken between December 2019 and March 2020.

### 2.4 Data analysis

Inductive thematic analysis was used based on its epistemological and theoretical flexibility.^22^ Transcripts were read through to familiarize with the corpus of the data and then analysed by one researcher using open coding and constant comparison, informed by the field notes. A framework of themes was developed from the analysis, together with a code book, and used to structure verbatim responses onto a spreadsheet. Where data did not fit generated themes, new codes were developed or existing ones were revised until all data were coded. This reflexive process^22^ was undertaken independently by one researcher supplemented by collaborative discussion with the second researcher to reach consensus and for confirmation of themes. The study researchers also used these discussions to consider the role of reflexivity and how to minimize the influence of their beliefs, personalities and experiences on the data analysis and interpretation.^23^

## 3 RESULTS

Fifteen patients participated in interviews (Table 1).

**Table 1:** Participant characteristics

Characteristic	Participants (*n* = 15)
Age (years)	
≤49	1
50–64	7
≥65	7
Gender	
Female	7
Male	8
Ethnicity	
White British	4
Asian British	3
Black British	8
Time on haemodialysis therapy (years)	
<2	3
2–4	5
5–9	4
≥10	3

Fourteen participants were on HD. One participant had discontinued HD after receiving a kidney transplant and two others had restarted HD treatment after kidney transplant failure. All participants receiving HD were attending their dialysis unit three times a week for 4 h at a time on a dialysis machine.

The themes and subthemes extracted through analysis are summarized and described in Table 2.

**Table 2:** Summary of overarching themes and subthemes

Overarching themes	Subthemes
1. Perceptions of the offer of activity participation and influences on take‐up	Relief from boredom Opportunity to learn something new Choice of activity
2. Experience of undertaking an activity or activities	Diversion from receiving haemodialysis Sense of achievement More positive self‐identity Increased confidence and motivation Therapeutic talking relationship
3. Suggested improvements	Peer stories and peer support Timing of activity sessions Matching tutors and activities to patient profile

### 3.1 Perceptions of the offer of activity participation and influences on take‐up

#### 3.1.1 Relief from boredom

For many participants, the offer of undertaking an activity was perceived as a welcome relief from boredom. Some participants described time as passing slowly during HD. Others talked about how time spent on HD was ‘empty’ time, or ‘time away from living’. Participants' accounts were often permeated by expressions of frustration, anger or distress. In a few cases, participants reported that because of the boredom, they would have been willing to take up almost any activity.

*Look being incarcerated here is tedium. Anything that breaks up tedium is a welcome relief. If it includes a bit of creativity, if it involves a bit of education, all the better*. (Patient 7)

#### 3.1.2 Opportunity to learn something new

The anticipated learning benefits of taking up an arts or creative living activity were an attraction for several participants. For example, one participant mentioned the perceived appeal of learning new skills. Another participant talked enthusiastically about gaining new knowledge and another about the opportunity for personal growth. Some participants stressed how important and beneficial it was to them to embrace ‘active’ as opposed to ‘passive’ activities such as watching TV or sleeping while dialysing. They talked about the value of keeping positive and engaged. A few participants were keen to make positive comparisons between themselves and others. They perceived engagement in the programme to reinforce their self‐image as more active, alert and involved than others receiving dialysis who spent their time more passively.

*It's good to keep your mind active… Three days a week for us, in each session just lying here would not be good at all. It just makes you more alive, more with things, you take more interest in life*. (Patient 1)

#### 3.1.3 Choice of activity

Providing patients with a choice of activities was considered to facilitate uptake in different ways. First, some participants reported that being offered a choice had enabled them to select an activity of individual appeal.

*Each individual has their own interests… S/he [tutor] had so many [activities] and I said I'm interested in this? Do you teach that? and she said ‘yes’… I went for it*. (Patient 12)

Second, several participants reported switching between activities. In some cases, participants said they changed their activity because they did not enjoy their first choice, and in other cases because their chosen activity sessions were completed or no longer available and another option was on offer. Additionally, some participants expressed appreciation of the offer of choice as it demonstrated recognition that the HD community is made up of individuals, each with their own interests, not one homogenous group.

*Everyone's got their choices. I think it's up to them really what they want to do… it's the patient's choice*. (Patient 5)

### 3.2 Experience of undertaking an activity or activities

#### 3.2.1 Diversion from receiving HD

Participating in an arts or creative living activity was particularly valued for providing a positive distraction from HD. Some participants talked about being so focused on the activity that their mind was diverted from the problems associated with receiving HD. Others reported that time passed much faster. A few participants reported that doing an activity had been emotionally therapeutic. They described themselves as being distracted from their problems related to HD and felt better able to cope with the treatment.

*You're not thinking about your dialysis, you're blanked out and thinking about what you're doing. It's amazing how focused you are… if you're busy doing nothing you have problems… It gives you the skills of how you can cope rather than just moan about it*. (Patient 4)

Experiencing these benefits led some participants to describe a sense of positive anticipation about attending HD sessions in which they knew they would be doing their chosen activity.

*It made me more happier for doing something. I got excited every Monday when s/he came I got something to do, at least it let the time pass quickly*. (Patient 13)

#### 3.2.2 Sense of achievement

Many participants positively described feelings of a sense of accomplishment resulting from engaging in an activity. No matter how much knowledgeable guidance they reported having from a tutor, perceiving themselves to be an agent of their own achievement and success was an important benefit. Some participants described mastering a new skill, others talked about discovering a talent they never knew they had or learning to do something never done before. Furthermore, such achievements were often reported with some incredulity; participants used words such as ‘amazed’, ‘shocked’ and ‘surprised’ to describe what they had achieved: ‘I didn't think I could ever like do something as good as that… I surprised myself’ (Patient 8).

Achievement was also reported in relation to using time on HD to do something constructive rather than it being ‘wasted’ time. Some participants described a sense of fulfilment about using dialysis time to learn or make something instead of sleeping.

*Coming here for these kind of hours, if you learn something out of it, it's good. I've enjoyed it. When they're [the tutors] here at least I've learnt something. Not coming to sleep, learn something*. (Patient 12)

Celebrating their achievements was reported by several participants as being particularly rewarding. Some participants mentioned feeling motivated because of receiving the approbation of their tutor. Others spoke about their pride and pleasure of having the product of their activity exhibited publicly in the hospital site.

#### 3.2.3 More positive self‐identity

Several participants reported experiencing a more positive sense of self and identity, beyond that of a renal patient receiving HD. The participants described this enhanced sense of self as resulting from positive changes in family and friends' attitudes to them following talking about what they were achieving in the activity or showing the output.

*People they look at you and they kind of make dialysis define you… for instance, when I finished [activity], I took [the activity work] and I showed my partner and even he was quite surprised at my skill… I felt like for once he looked at me as someone that's capable of making something or doing something outside of being here [the dialysis unit]*. (Patient 15)

#### 3.2.4 Increased confidence and motivation

Engaging in an arts or creative living activity was also considered to be beneficial to participants' confidence and motivation. Some participants spoke about feeling more empowered as a result of mastering a new activity. For example, a male participant described how learning something new had given him more confidence in social situations:

*It did make me feel in some situations maybe a little more confident… Initially it was just about the [activity] and then I suppose after you learn that you can manage, you think well I'm not that stupid*. (Patient 6)

Other participants reported how the process of learning or creating had generated new possibilities and opportunities such as joining other local educational classes, restarting hobbies and pursuing employment.

*I'm always failing I never push myself. If I can make an object, I don't see why I can't do what I want to do in future*. (Patient 13)

#### 3.2.5 Therapeutic talking relationship

A few participants reported that part of the enjoyment of undertaking an activity was the opportunity to talk with someone (the tutor) while on HD. ‘It definitely made the mood a little bit lighter… just looking forward to speaking to somebody else’ (Patient 6). For these participants, talking seems to have been therapeutic for a variety of different reasons: break from isolation; association with a human rather than a machine; discussion on topics other than dialysis; and an opportunity to discuss emotions.

### 3.3 Suggested improvements

Participants suggested several improvements for the arts programme. These suggestions were associated with more negative aspects of their experience of the programme. The suggested improvements were reported in three themes:

#### 3.3.1 Peer stories and peer support

Peer patient promotion of the benefits of participation was a frequent suggestion to improve uptake of the programme. While appreciative of the promotion by tutors, several participants explained that patients who shared the lived experience of HD could provide a truer and more inspiring story of undertaking an activity while dialysing.

*Maybe they should get other patients who have done it before, maybe they should speak to the patients who are dialysing and explain to them it's a good thing… they have something in common so they will understand where you're coming from*. (Patient 13)

Some patients suggested that exhibiting their work on the dialysis unit walls might support their peers who knew less about arts or creative activities to understand what was involved and what might be achieved.

#### 3.3.2 Timing of activity sessions

Several participants suggested that there needed to be more choice as to when activity sessions were undertaken to fit with the physical and emotional stresses of coping with HD. Some participants reported that there were occasions when they were not emotionally and/or physically receptive to engaging in the activity.

*I might come with a headache or I might not feel too well and like it's the [activity] day today and because I get my joy from the activity you feel obligated to*. (Patient 15)

#### 3.3.3 Matching tutors and activities to patient profile

Some participants, particularly those identifying as Black British, argued that an increase in take‐up could be achieved by expanding the activity appeal to a wider socioeconomic range of patients. To achieve this, they felt that there needed to be a better match of tutors and activities to the profile of patients in the dialysis units, in particular, taking into account age and ethnicity.

*It will depend what the community of patients are. Like the satellite unit at [unit name], in the group that I am in, a lot of the patients are elderly, some of them have a language barrier to understanding… you might basically have a visit there to see the demographic of the people as to what they might or could be offered*. (Patient 6)

## 4 DISCUSSION

This qualitative study identified positive views from patient participants to the experience of engaging in their chosen arts or creative living activity while receiving HD. Positive experiences of patients on HD participating in arts programmes have been reported in other studies.^9^, ^10^, ^12^, ^13^, ^15^ As with Rowe et al.,^13^ our findings indicated that a main patient explanation for the positive experience was diversion from the tedium of receiving HD as well as a motivating factor in activity take‐up. However, in this study, participants also discussed their positive experience in the context of feeling a sense of achievement in what they accomplished and in discovering unknown skills and talents. They perceived the activities to have therapeutic value not only during HD but also more generally for living as a patient on HD. Longer‐term benefits were reported as experiencing a more positive self‐identity leading to increased confidence and motivation to take part in other activities and interests. To our knowledge, these findings have not been reported before.

Our appraisal of published evidence identified that only Rowe et al.^12^ posited any explanatory theory for the positive responses of patients, an analysis confirmed in a recent realist review.^24^ One possible theory suggested by Rowe et al.^12^ was that of Csikszentmihalyi's^25^ concept of ‘flow’. Csikszentmihalyi argued that optimal human experience was achieved when people were in flow: A state of concentration so absorbing that they described time as passing much faster. The conditions identified as likely to create a state of flow were as follows: The person perceived there to be an opportunity for action (challenge); for stretching their capabilities; and the likelihood of learning new skills. While we concur that this theory has explanatory value for some of the patient‐reported positive experiences in this study, we suggest that there may be three alternate explanatory mechanisms that require future investigation. We discuss these in turn.

Our participants' positive experiences, beyond the immediate absorption of the ‘challenging’ activity, could be explained by considering aspects of cognitive adaptation. Taylor^26^ argued that people faced with personally threatening events adjusted and coped through three processes: a search for meaning in the experience; attempts at mastery over the event or more generally in life; and attempts to build self‐esteem. These processes were described with reference to patients with cancer, although they are likely to apply to patients with other chronic or fatal diseases including patients with ESKD. There has long been evidence of the continuous coping and adjustment challenges faced by patients with kidney failure and the impact of dialysis on self‐esteem, particularly in relation to feeling less capable and productive than before kidney failure.^27^, ^28^ We theorize that the accounts of more positive self‐identity and increased confidence provided by our participants reflected changes in their self‐esteem contributing to overall well‐being. Several participants also reported distinguishing themselves favourably from other dialysis patients who they perceived as more passive and disengaged because they had chosen not to participate in an activity. Making active self‐enhancing comparisons with others in a similar position, to compare positively downwards, was described by Taylor^26^ as a method of bolstering self‐esteem. It is unknown the extent to which the patient group who accepted the offer of engaging in an activity, and volunteered for the study, were already active in the three areas of cognitive adaption that Taylor^26^ described. This requires further investigation in future studies.

An alternative explanatory theory can be found in Goffman's^29^ theory of stigma. A stigmatizing condition is characterised by discrediting attributes being assigned to individuals by others. Our findings showed that enhanced self‐worth for some participants was linked to positive changes in family members' perceptions of them consequent to engaging in an activity. Changed perceptions appeared to be an affirmation of their identity as one separate from that of a patient on dialysis. We theorize that in undertaking and engaging in the arts and creative activities, the participants disrupted the assignment of discrediting attributes by others and lessened the stigmatizing effect of receiving HD.^29^ This also requires further investigation and testing.

A third potential explanatory theory for the positive experiences described by our participants could be the reported therapeutic conversations with some tutors. This programme provided one‐on‐one tuition at the bedside rather than group activities (as described by most other studies^9^, ^11^, ^12^, ^13^, ^14^). There is evidence that cognitive behaviour therapy has beneficial effects for dialysis patients diagnosed with depression.^30^ The influence on mood and well‐being of one‐on‐one interactions compared to group activities, which encourage more social interaction, is another area for further examination.

This study also reports that for many participants, having choice was important as to which arts and creativity living activity they engaged in. This is congruent with findings from previous literature on the importance for patients with kidney failure of having choice and control in relation to the timing and delivery of dialysis^31^ and support interventions.^32^ Greater diversity in activity choice was also recommended, matched to the interests of the socioeconomic profile of dialysis unit patient populations. Participants identifying as Black British particularly emphasized that this improvement to the programme was needed to broaden its appeal. Interestingly, this was the only notable difference in terms of age, ethnic background or dialysis experience in participant responses to the intervention. Additionally, the participant advocacy of peer promotion of the uptake of arts and creative activities is in keeping with other studies reporting the value of peer support for patients with shared lived experience of dialysis.^32^

HD is a challenging, time‐consuming therapy due to the lengthy time periods spent on the dialysis machine and the time commitment required to travel to and from dialysis centres. Time spent on machines is viewed as redundant and frustrating. This leads to significant challenges in adherence to the therapy. Poor compliance to HD leads to significant morbidity and mortality as well as increased burden on the NHS. Engaging in a pleasurable activity during dialysis provides a potential incentive to increase dialysis adherence. More broadly, the study findings and theoretical mechanisms for such findings are likely to be of value to policy makers at the national level concerned about how best to support the mental health and well‐being of the increasing numbers of people living with chronic and end‐stage health conditions, including through participation in the arts.^7^ They will also contribute to the evidence base for funding arts‐based programmes for these patient groups. The study findings will further be of value to clinicians and decision makers considering specifically how to support the therapeutic needs of patients receiving HD and the contribution that arts programmes can make.

### 4.1 Strengths and limitations

A major strength of the study was PPIE involvement in the design, which prompted and guided consideration of context in understanding participants' experience of undertaking an arts and creative living activity while receiving HD. Theoretical framing of the findings contributed towards an understanding of participants' positive experiences. The study has some limitations. Participants of only one arts programme were recruited, a limitation that can only be addressed by involving more renal centres. However, there are few opportunities currently for patients to participate in such programmes in renal centres in the United Kingdom,^22^ and the international experience suggests precarious programme funding. The study used one female interviewer with previous experience of interviewing patients on dialysis, which may have influenced their approach. However, the team approach to analysis and drafting the paper helped mediate against a single interpretation. The study was not funded to translate study materials into different languages or conduct the interviews in languages other than English. Nevertheless, the study participants were diverse in terms of age, ethnicity and dialysis experience, thus increasing the potential transferability of our findings. The study did not actively seek to ask eligible patients who had not participated in the arts programme their reasons for nonparticipation, as there were no records of who had been offered the programme and declined. However, to mitigate this limitation, study participants were asked their views on why other patients might have chosen not to participate in the programme.

## 5 CONCLUSION

This study reports positive patient experiences of engaging in a chosen arts or creative living activity while receiving HD, which in turn had a positive influence on the dialysis experience. Irrespective of the activity chosen, participation was perceived to have therapeutic value through improving mood and well‐being as well as generating longer‐term benefits associated with increased confidence and enhanced self‐esteem. This evidence will be of value to policy makers, service commissioners and health professionals considering introducing or supporting such programmes. Our analysis and interpretation suggest theoretical mechanisms for these findings and as such adds both new knowledge to the evidence base and theoretically informs future studies of impacts and outcomes.

## CONFLICT OF INTERESTS

The authors declare that there are no conflict of interests.

## ETHICS STATEMENT

This study was approved by a UK NHS Research Ethics Committee (Reference number 19/WA/0324). Written informed consent was obtained from each interview participant.

## AUTHOR CONTRIBUTIONS

Vari M. Drennan and Francesca Taylor conceptualized the study and were responsible for funding acquisition. Vari M. Drennan and Francesca Taylor formulated the methodology. Francesca Taylor, Jeunita Jones, Marie‐Louise Turner and Joyce Popoola contributed to the investigation. Francesca Taylor and Vari M. Drennan carried out the formal analysis. Francesca Taylor was responsible for the first draft of the manuscript; and all authors are in agreement of the final manuscript and its revisions.

## Supporting information

Supporting information.

## Data Availability

Deidentified interview data sets analysed in the current study are available from the corresponding author on reasonable request.
