# Supplementary material for: ‘It gives you the skills of how you can cope’: Exploring the self‐reported experience of patients receiving in‐centre haemodialysis on participating in chosen art activities
Source: Health Expect. 2021 Aug 11;24(6):1979–87. doi: 10.1111/hex.13337 (PMC8628595; doi:10.1111/hex.13337)
Supplement: Supplementary file 1 — Supporting information. [file HEX-24-1979-s001.docx]

**Supplementary file 1**

**‘It gives you the skills of how you can cope’: exploring the self-reported experience of patients receiving in-centre haemodialysis on participating in chosen art activities.**

**Checklist for standards for reporting qualitative research** ^1^

| **Item** |  | **Description** | **Page number in manuscript** |
| --- | --- | --- | --- |
| 1 | TITLE | Concise description of the nature and topic of the study. Identifying the study as qualitative | 1 |
| 2 | ABSTRACT | Summary of key elements using the format of the publication; | 1 |
| 3 | Problem Formulation | Description and significance of the problem/phenomenon studied; review of relevant theory and empirical work; problem statement. | 2, 3 |
| 4 | Research question | Purpose of the study and specific objectives or questions. | 3 |
| 5 | Qualitative approach and research paradigm | Qualitative approach; identifying the research paradigm (e.g., post-positivist, constructivist/interpretivist) | 3 |
| 6 | Researcher characteristics & reflexivity | Researchers’ characteristics that may influence the research. | 4, 10 |
| 7 | Context: | Setting/site and salient contextual factors; rationale. | 3 |
| 8 | Sampling strategy | How and why research participants, documents, or events were selected; criteria for deciding when no further sampling was necessary (e.g., sampling saturation); rationale. | 3, 4 |
| 9 | Ethical issues pertaining to human subjects | Documentation of approval by an appropriate ethics review board and participant consent, or explanation for lack thereof; other confidentiality and data security issues. | 11 |
| 10 | Data collection methods: | Types of data collected; details of data collection. | 4 |
| 11 | Data collection and technologies | Description of instruments (e.g., interview guides, questionnaires) and devices (e.g., audio recorders) | 4 |
| 12 | Units of study | Number and relevant characteristics of participants, documents, or events included in the study; level of participation. | 4, 5 |
| 12 | Data processing | Methods for processing data prior to and during analysis, including transcription. | 4 |
| 14 | Data analysis: | Process by which inferences, themes, etc. were identified and developed, including the researchers involved in data analysis | 4 |
| 15 | Techniques to enhance trustworthiness | Techniques to enhance trustworthiness and credibility of data analysis, rationale. | 4 |
| 16 | Synthesis and interpretation | Main findings (e.g., interpretations, inferences, and themes. | 4-8 |
| 17 | Links to empirical data | Evidence (e.g., quotes, field notes, text excerpts, photographs) to substantiate analytic findings. | 5-8 |
| 18 | Integration with prior work, implications, transferability, and contribution(s) to the field | Short summary of main findings, explanation of how findings and conclusions connect to, support, elaborate on, or challenge conclusions of earlier scholarship; discussion of scope of application/generalizability; identification of unique contribution(s) to scholarship in a discipline or field. | 8-10 |
| 19 | Limitations | Trustworthiness and limitations of findings | 8 |
| 20 | Conflicts of interest | Potential sources of influence or perceived influence on. | Title page |
| 21 | Funding | Sources of funding and other support; role of funders in data collection, interpretation, and reporting. | Title page |

^1^ O'Brien BC, Harris IB, Beckman TJ, et al. Standards for reporting qualitative research: a synthesis of recommendations. *Acad Med*. 2014;89(9):1245-1251. doi:10.1097/ACM.0000000000000388
